# PHYSICAL ACTIVITY LEVELS ARE LOW IN THE DAYS, WEEKS, AND MONTHS FOLLOWING DYSVASCULAR MAJOR LOWER LIMB AMPUTATION

**DOI:** 10.2340/jrm.v58.44666

**Published:** 2026-05-05

**Authors:** Aniek M. KOLEN, Leonie A. KROPS, Harriët JAGER-WITTENAAR, Herwin HOREMANS, Johannes B. J. BUSSMANN, Martijn L. DIJKSTRA, B. Paul J. A. KELLER, Jacques OSKAM, Bastiaan VIERHOUT, Jean-Paul P. M. DE VRIES, Laurens VAN WALRAVEN, Wim P. KRIJNEN, Jan H. B. GEERTZEN, Rienk DEKKER

**Affiliations:** 1 University of Groningen, University Medical Center Groningen, Department of Rehabilitation Medicine, Groningen; 2 Hanze University of Applied Sciences, Research Group Healthy Ageing, Allied Health Care and Nursing, Groningen; 3 Radboud university medical center, Department of Gastroenterology and Hepatology, Dietetics, Nijmegen, The Netherlands; 4 Vrije Universiteit Brussel, Faculty of Physical Education and Physiotherapy, Department Physiotherapy, Human Physiology and Anatomy, Research Unit Experimental Anatomy, Brussels, Belgium; 5 Department of Rehabilitation Medicine, Erasmus MC, University Medical Center Rotterdam, Rotterdam; 6 Department of Surgery, Division of Vascular Surgery, University Medical Center Groningen, University of Groningen, Groningen; 7 Martini Hospital, Department of Vascular Surgery, Groningen; 8 Isala Hospital, Department of Surgery, Division of Vascular Surgery, Zwolle; 9 Department of Surgery, Treant Hospital, Emmen; 10 Medical Center Leeuwarden, Department of Vascular Surgery, Leeuwarden, The Netherlands

**Keywords:** amputation, determinants, exercise, peripheral arterial disease, physical activity

## Abstract

**Objective:**

To determine physical activity levels and their associated factors post-dysvascular major lower limb amputation.

**Design:**

Multicentre longitudinal observational study.

**Subjects:**

Eighty-one adults with dysvascular major lower limb amputation.

**Method:**

Physical activity was assessed via questionnaire (adapted-SQUASH: total min, moderate/vigorous min, MET-min/week), self-reported adherence to Dutch physical activity guidelines, and accelerometer (Activ8: min, bouts, fragmentation) at < 12 days, 5 weeks, 6 months, and 9 months post-amputation. Associated factors were assessed using mixed model analysis.

**Results:**

Median [interquartile range] questionnaire-assessed activity increased: 240 [90;600], 525 [245;880], 678 [334;1176], and 885 [525;1733] min/week (*p* = 0.004). Dutch guideline adherence was 16%, 55%, 47%, and 27%, respectively. Accelerometer-derived activity was 5 [5;9], 26 [13;50], 50 [33;64], and 54 [44;67] min/day. Number of active bouts ranged from 0.0 [0.0;0.0] to 2.0 [1.0;3.0] per day, with fragmentation between 0.0 [0.0;0.0] and 0.4 [0.4;0.5]. Questionnaire-assessed physical activity was negatively associated with age (β = –16 min/week, *p* = 0.009) and undernutrition (β = –270 min/week, *p* = 0.013), and positively with grip strength (β = 14 min/week, *p* = 0.014).

**Conclusion:**

Physical activity levels increase but remain low post-lower limb amputation. Age, undernutrition, and grip strength are associated with physical activity. These findings underscore the need for targeted physical activity interventions for individuals post-amputation, to optimize health and clinical outcomes.

## LAY ABSTRACT

Physical activity is known as an important preventive strategy for health and well-being, which is also the case in individuals with disabilities. However, staying physically active can be challenging for people with a lower limb amputation, for example because of pain and mobility difficulties. This study followed 81 adults during the first 9 months after amputation due to vascular disease to gain insight into how active they were and what factors were associated with their activity levels. Physical activity was assessed using questionnaires and a wearable device at 4 time points: shortly after amputation, and at 5 weeks, 6 months, and 9 months. Activity levels increased over time but remained low. Older age and undernutrition were associated with lower activity, while higher grip strength was associated with higher activity. These findings highlight the need for targeted rehabilitation programmes that support physical activity after amputation, which may help to improve recovery and long-term health outcomes.

In the general population, physical activity (PA) has well-established benefits for physical and mental health, including reduced risk of cardiovascular disease and lower all-cause mortality rates (1–3). However, for individuals recovering from a dysvascular major lower limb amputation (LLA), engaging in PA may be challenging due to factors such as difficulties with mobility and pain (4), potentially negatively affecting health and clinical outcomes.

While PA likely plays a key role in rehabilitation, previous research on the level of PA post-LLA primarily focused on the long term, e.g., multiple years post-LLA, on specific subgroups, e.g., people with unilateral transtibial amputation or prosthesis users, or on those with various aetiologies of LLA (5–11). As a result, to the best of our knowledge, the level of PA during the first days, weeks, and months following dysvascular major LLA is unknown. Moreover, the factors that influence PA in the relatively early phase post-LLA are unknown.

Insight into short-term PA and its associated factors is needed to explore whether it should play a role in the development of interventions in order to optimize patient and clinical outcomes. Therefore, the current study aimed to assess PA levels on various measurement occasions following dysvascular major LLA, and to identify factors associated with PA.

## METHODS

The current study applied a prospective longitudinal observational cohort design, in which usual care was provided. The study was performed according to the Declaration of Helsinki. This study was part of a larger study on nutrition and PA post-dysvascular major LLA, which was registered on ClinicalTrail.gov (NCT05747066). Medical ethical approval for this study was granted by the Medical Ethical Committee of the University Medical Center Groningen (2022/387). This study was reported following the reporting guidelines STrengthening the Reporting of OBservational studies in Epidemiology (STROBE) (12).

### Participants

People who have undergone a dysvascular major LLA were recruited from February until December 2023, in 6 hospitals in the Northern Netherlands: University Medical Center Groningen, Isala Hospital Zwolle, Martini Hospital Groningen, Medical Center Leeuwarden, Treant Hospital Emmen, and Ommelander Medical Group Scheemda. Inclusion criteria were: having undergone a dysvascular major LLA (i.e., Syme amputation or more proximal level), being ≥ 18 years old, and being physically, mentally, and communicatively able to participate. People were excluded if they required a reamputation during the study period or had a severe malabsorption disease. All participants provided written informed consent before voluntarily participating in the study. Participants received a €10 voucher per completed measurement occasion for their time investment.

### Data collection

This study consisted of 4 measurement occasions: T1 being between 2 and 12 days post-LLA (target 4–5 days post-LLA), T2 at 5 (range 3–7) weeks post-LLA, T3 at 6 (range 5–7) months post-LLA, and T4 at 9 (range 8–10) months post-LLA. The T1 measurement could be missed due to the participant’s physical condition, the consideration time needed for participants to decide on participation, and logistic challenges. T2 was chosen as the time point at which sutures have typically been removed. The time point of T3 was selected as most people have left the in/outpatient rehabilitation facility after 6 months, and T4 as the time point after which prosthesis use is expected to remain fairly constant.

**Fig. 1:**
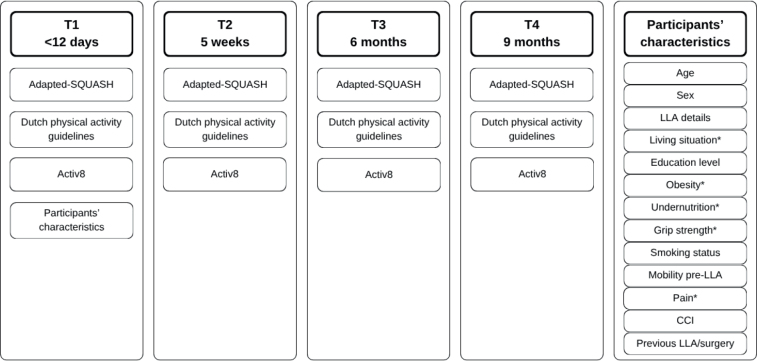
Data collection on every measurement occasion. In any case where the T1 measurement was missed, participants’ characteristics were collected at T2. LLA: lower limb amputation; CCI: Charlson Comorbidity Index. *Variable was assessed on every measurement occasion.

Fig. 1 provides an overview of the types of measurements taken per measurement occasion. The measurements were performed by a researcher (AK) or trained senior year or graduate student in Dietetics or Human Movement Sciences, at the participant’s place of living. Data were entered and managed using REDCap (13, 14). Random checks (≥ 10%) were conducted to identify input errors in the database.

### Adapted-SQUASH

The Adapted- Short QUestionnaire to ASsess Health-enhancing physical activity (Adapted-SQUASH) is a Dutch 19-item self-reported recall questionnaire designed to assess PA among persons with disabilities (15). The participant filled in the number of days, average hours and minutes per day, and perceived intensity (light, moderate, vigorous) of different types of activities in each setting in the previous week. The settings included commuting activities, work/school activities, household tasks, leisure activities, and sports.

The minutes of activity per week were determined by multiplying the administered average number of days and average duration of the activity. To assess activity intensity, the perceived intensity of each activity was combined with its corresponding metabolic equivalent of task (MET) value, based on the Ainsworth compendium of physical activities (16) and compendium of PA for wheelchair users (17) (Table SI). To calculate the total activity score, intensity levels were categorized as follows: light (< 4 MET for individuals aged 18–55 years, < 3 MET for those over 55 years at moment of LLA), moderate (4–6.5 MET for ages 18–55 years, 3–6 MET for those over 55 years at moment of LLA), and vigorous (> 6.5 MET for ages 18–55 years, > 6 MET for those over 55 years at moment of LLA), and multiplied by the total number of minutes of activity per week. Outcomes included total number of minutes of PA per week, as well as minutes of light, moderate, and vigorous activity per week, both overall and by setting, and the total activity score. The Adapted-SQUASH has good reliability for total minutes of activity per week and total activity score, and validity comparable to other PA questionnaires when compared with accelerometer-derived PA (15).

### Meeting Dutch PA guidelines

Participants were asked whether they met the Dutch PA guidelines (yes/no), which include a minimum of 150 minutes of moderate to vigorous PA activity per week, and muscle and bone strengthening activities twice a week (18).

### Activ8

The Activ8 professional GEN1 is a single-unit PA monitor with a triaxial accelerometer (raw sample frequency 12.5 Hz; 2M Engineering, Valkenswaard, The Netherlands) and has compact dimensions of 30x32x10 mm and a weight of 20 g. It is a valid instrument to quantify lying/sitting, standing, walking, cycling, and running in people who have undergone a unilateral LLA who wear a prosthesis (19), healthy adults (20), and hospitalized patients (21), and is valid for distinguishing between active and non-active wheeling (22, 23). Epoch length was set at 15 s. The Activ8 was packaged in a waterproof pouch and taped by the researcher to the ventral side of the participant’s intact upper leg, 10 cm distal from the groin. In the case of bilateral LLA, the Activ8 was attached on the less recently amputated side. For participants who used a manual wheelchair, an Activ8 device was attached closest to the centre of the wheel and another Activ8 in a strap on the participant’s wrist. The latter sensors allowed for differentiation of self-performed wheelchair driving from somebody else pushing the wheelchair, which was not counted as the participant’s PA. Three Activ8 devices were attached on the leg, wheel, and wrist, in any case where the participant had a stand/walk function, used a manual wheelchair, and sometimes somebody else pushed the wheelchair. The Activ8 was worn for 2 to 7 consecutive days, 24 h per day, including sleeping, bathing, and swimming. The goal, not a prerequisite, was to wear the device for at least 4 days. The criteria for wearing an Activ8 included having intact skin, no allergy to the tape, and no presence of delirium or other cognitive issues that might increase the risk of removing and not returning the device. Participants returned the Activ8(s) by post or the devices were picked up from home by the researcher.

The raw Activ8 data are in-sensor converted to postures and movements at a resolution of 1.56 Hz (24 samples). After data download, a custom-made Matlab script was used to determine wheelchair activity and to summate all postures and movements (sitting/lying, standing, walking, cycling, running, wheeling, and manoeuvring [minor wheel movements]) over the 15 s epoch. Outcome measures were duration of PA (summed duration over walking, cycling, running, wheeling, and manoeuvring), number and duration of active bouts and fragmentation of active bouts (duration of active bouts/number of active bouts). Bouts were defined based on 1-min intervals (sum of four 15 s intervals) of the following 3 types: active (≥ 80% of time spent walking, running, cycling, wheeling, or manoeuvring), sedentary (≥ 90% of time spent sitting or lying), or neutral (time intervals that were neither active nor sedentary). Active bouts were defined as a sequence of consecutive active intervals, allowing isolated neutral intervals, provided that at least 70% of the total bout duration consisted of walking, cycling, running, wheeling, and manoeuvring. Sedentary bouts were defined as sequences of consecutive sedentary intervals, allowing isolated neutral intervals, where at least 90% of the time was spent sitting or lying. Prolonged active bouts were defined as active bouts lasting longer than 10 min, while prolonged sedentary bouts were defined as sedentary bouts lasting longer than 30 min. Furthermore, data from the Activ8 placed on the leg were used to measure overall counts per minute, walking counts, and active counts, with active counts including the sum of the counts measured during walking, cycling, and running.

### Participants’ characteristics

Participants’ characteristics were obtained with a self-reported questionnaire or determined by the researcher, including age at the moment of LLA, sex (male, female), characteristics of LLA (uni- or bilateral, level(s) of LLA(s)), comorbidities (self-reported Charlson Comorbidity Index), any pain (Visual Analogue Scale [VAS] of average and maximal pain in last week), obesity (BMI ≥ 30 kg/m^2^ – corrected for missing limb [24–26]), undernutrition (Patient-Generated Subjective Global Assessment [27, 28]); dichotomized into: well-nourished vs moderate/suspected undernutrition or severely undernourished), smoking status (never, previous, current), educational level (categorized as low: elementary or high school; moderate: vocational college; or high: [applied] university), living situation (type of facility or home, alone or with others), mobility pre-LLA (independent outdoor ambulator, independent indoor ambulator, aid depended, wheelchair, or bedridden), the number of previous revascularization procedures and/or LLA(s), and the K-level at T3 and T4. Moreover, hand grip strength was assessed using a hand dynamometer (Jamar, TEC Inc, Clifton, NJ, USA). Participants sat with their elbow at 90° without support. The dominant hand was tested 3 times, and the highest value was used.

### Data analysis

Descriptive statistics were used to summarize participant characteristics and PA levels at every measurement occasion and between measurement occasions. Continuous variables were presented as mean and standard deviation, or as median and interquartile range [Q1; Q3], depending on normality of the underlying distribution. Categorical variables were presented as frequencies and percentages.

To investigate the change over time for PA levels, a mixed model analysis with restricted maximum-likelihood and unstructured correlation structure was used, with measurement occasion as fixed time factor and a random intercept for each participant to account for individual specific levels. The following covariates were used: age, sex, amputation level (low, high), Charlson Comorbidity Index without age, average pain, obesity, and undernutrition. To evaluate pairwise differences between measurement occasions, planned linear contrasts were used with the general linear hypothesis testing framework in the R multcomp package (R Foundation for Statistical Computing) and computed adjusted *p*-values (29, 30).

To investigate the determinants for PA, a univariate linear mixed model analysis was conducted to identify potential predictors for inclusion in the multivariate model. Determinants with a *p*-value < 0.1 in the univariate analysis were included in the multivariate model, controlled for age and sex. Moreover, variables were selected using a stepwise algorithm guided by the Akaike Information Criterion (31).

The significance of fixed effects of measurement occasion was tested by likelihood ratio. The results were considered statistically significant at a *p*-value < 0.05. The validity of the conclusions on the estimated effects was checked by a residual analysis including Cook’s distance. Analysis was performed in the statistical programming language R (v4.4.0) (32), using the lmer function of the lme4 package (33).

## RESULTS

### Participants

**Fig. 2:**
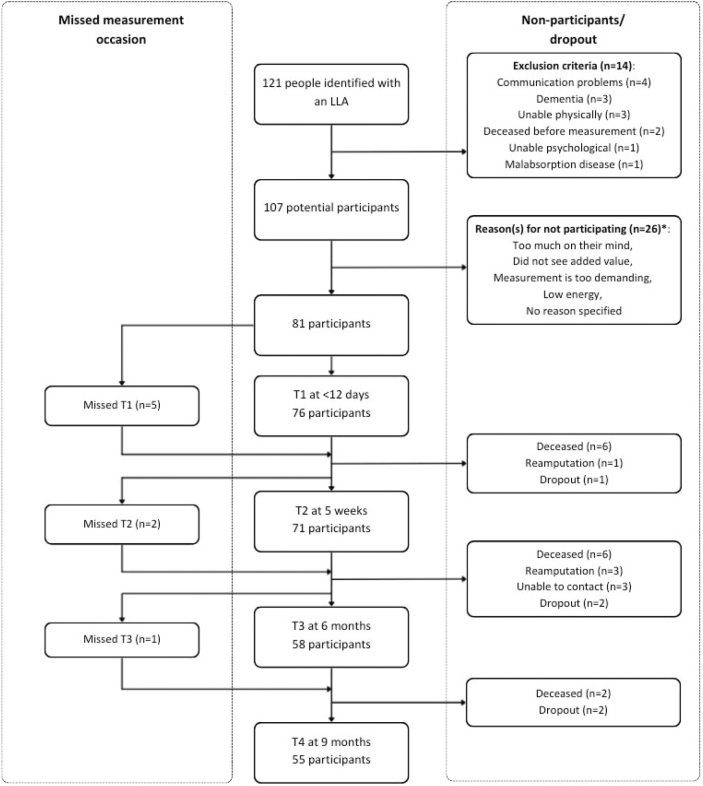
Illustration of recruitment, displaying the number of people identified, potential participants, enrolled participants, and number of participants per measurement occasion. *One or more reasons per potential participant for not being willing to participate.

A total of 81 people with dysvascular major LLA (76% of eligible individuals) participated in the study (Fig. 2). The average age at the time of LLA was 69.7 (10.5) years, with 59 participants (73%) being male. The majority had a unilateral transtibial amputation, and the Charlson Comorbidity Index was 6.1 (2.3), or 3.6 (2.0) when not considering age for calculating the Charlson Comorbidity Index (Table I). T1, T2, T3, and T4 were performed 4.7 (1.8), 36 (3.9), 180 (10), and 274 (9.4) days post-LLA, respectively.

**Table I:** Baseline characteristics of participants (*n* = 81)

Age, years^a^, mean (SD)	69.7 (10.5)
Sex, male, *n* (%)	59 (73)
Amputation level, *n* (%)	
TT	59 (73)
KD	3 (4)
TF	19 (24)
Bilateral amputation, *n* (%)	13 (16)
TT	10
TF	3
Living situation pre-LLA, *n* (%)	
Home	75 (93)
Alone	24
Care facility	6 (7)
Living situation (T2; T3; T4), *n* (%)	
Home	22 (31); 45 (78); 48 (87)
Skilled nursing facility	41 (58); 3 (5); 0 (0)
Rehabilitation facility	1 (1); 0 (0); 0 (0)
Nursing home	3 (4); 9 (16); 6 (6)
Admitted to hospital	2 (3); 0 (0); 0 (0)
Other	2 (3); 1 (2); 1 (2)
Educational level^b^, *n* (%)	
Low	44 (54)
Moderate	22 (27)
High	14 (17)
Unknown	1 (1)
Obesity (T1; T2; T3; T4)^c^, *n* (%)	21 (28); 17 (24); 18 (31); 15 (27)
Undernutrition (T1; T2; T3; T4), *n* (%)	63 (84); 40 (56); 16 (28); 13 (24)
Grip strength (kg, T1; T2; T3; T4), mean (SD)	29 (11); 30 (11); 31 (12); 33 (10)
Smoking status, *n* (%)	
Current	23 (28)
Previous	37 (46)
Never	21 (26)
Mobility pre-LLA, *n* (%)	
Independent outdoor ambulator	24 (30)
Independent indoor ambulator	14 (17)
Aid dependent	38 (47)
Wheelchair or bedridden	5 (6)
Pain average (VAS 0–10) (T1; T2; T3; T4)^d^, median [IQR]	5.4 [2.3; 7.6]; 1.7 [0.0; 4.8]; 0.8 [0.0; 4.8]; 1.8 [0.0; 5.3]
Pain worst (VAS 0–10) (T1; T2; T3; T4)^d^, median [IQR]	9.7 [7.3; 10]; 4.6 [0.0; 8.6]; 2.5 [0.0; 7.2]; 4.8 [0.0; 8.1]
Charlson Comorbidity Index, mean (SD)	6.1 (2.3)
Charlson Comorbidity Index, without age, mean (SD)	3.6 (2.0)
Diabetes, *n* (%)	50 (62)
Type 2	44
Insulin dependent	40
Congestive heart failure, *n* (%)	29 (36)
Myocardial infarct, *n* (%)	13 (16)
Renal disease, *n* (%)	17 (21)
Undergoing dialysis	7
Chronic pulmonary disease, *n* (%)	10 (12)
Cancer, past or present, *n* (%)	14 (17)
Diabetes, *n* (%)	50 (62%)
Previous minor/major LLAs median [IQR]	0 [0; 1]
Previous vascular surgeries median [IQR]	2 [1; 4]
K-level, T3; T4, *n* (%)	
0	30 (52); 30 (55)
1	8 (14); 8 (15)
2	17 (29); 11 (20)
3	3 (5); 6 (11)
4	0 (0); 0 (0)

a Age at moment of LLA.

b Educational level: low: elementary or high school; moderate: vocational college; high: (applied) university.

c If bodyweight was unknown on measurement occasion, the bodyweight of the closest measurement occasion was used to calculate BMI.

d One participant did not score his/her pain.

SD: standard deviation; TT: transtibial; KD: knee disarticulation; TF: transfemoral; VAS: Visual Analogue Scale; LLA: lower limb amputation; IQR: interquartile range.

### Adapted-SQUASH

**Fig. 3:**
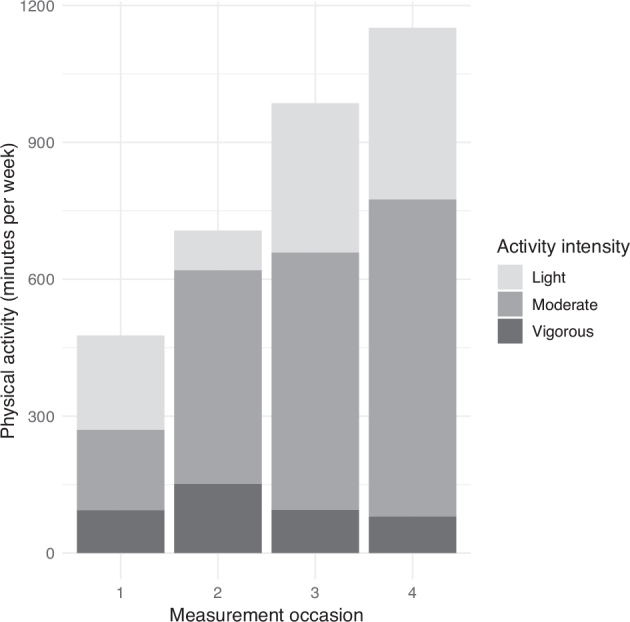
Questionnaire-assessed physical activity per week divided into light, moderate, and vigorous on the 4 measurement occasions, expressed in average minutes per week.

The median levels of questionnaire-assessed PA were 240 [90; 600], 525 [245; 880], 678 [334; 1176], and 885 [525; 1733] min/week, respectively, at T1, T2, T3, and T4 (*p* = 0.004). The median minutes per week spent on moderate/vigorous activity were 90 [18; 255], 420 [203; 748], 396 [159; 865], and 525 [183; 1260] min/week at T1, T2, T3, T4, respectively (*p* = 0.004) (Fig. 3). No moderate/vigorous activity was reported by 22%, 3%, 5%, and 6% of participants, respectively. Activity scores were 923 [443; 1943], 2310 [1373; 4145], 2540 [1144; 4943], and 2940 [1680; 6140] MET-min/week at T1, T2, T3, and T4, respectively (*p* = 0.006) (Table II).

**Table II:** Questionnaire-assessed physical activity per measurement occasion in total, per setting, intensity, mode, and frequency

Physical activity	T1 (*n* = 76) Median [IQR]	T2 (*n* = 71) Median [IQR]	T3 (*n* = 58) Median [IQR]	T4 (*n* = 55) Median [IQR]	*p*-value^a^	*p*-value^b^
**Total physical activity (min/week)**	240 [90; 600]	525 [245; 880]	678 [344; 1176]	885 [525; 1733]	< 0.001^§#>¥^	0.004^#>¥^
0 min/week	*n* = 8 (11%)	*n* = 2 (3%)	*n* = 3 (5%)	*n* = 2 (4%)		
Light (min/week)	80 [0; 375]	0 [0; 67]	195 [0; 420]	240 [30; 603]	< 0.001^#‡>¥^	< 0.001^†‡>¥^
0 min/week	*n* = 34 (45%)	*n* = 47 (66%)	*n* = 16 (28%)	*n* = 13 (24%)		
Moderate (min/week)	0 [0; 108]	210 [35; 615]	248 [101; 688]	420 [148; 1178]	< 0.001^†§#^	0.003^†§#^
0 min/week	*n* = 41 (54%)	*n* = 17 (24%)	*n* = 7 (12%)	*n* = 8 (15%)		
Vigorous (min/week)	3 [0; 105]	120 [35; 214]	48 [0; 105]	0 [0; 60]	0.034^>¥^	0.057
0 min/week	*n* = 38 (50%)	*n* = 13 (18%)	*n* = 22 (38%)	*n* = 33 (60%)		
Total activity score (MET-min/week)	923 [443; 1943]	2310 [1373; 4145]	2540 [1144; 4942]	2940 [1680; 6140]	< 0.001^†§#^	0.006^†§#^
**Commuting**						
Total (min/week)	0 [0; 0]	0 [0; 0]	0 [0; 0]	0 [0; 0]	FTC	0.391
0 min/week	*n* = 76 (100%)	*n* = 71 (100%)	*n* = 58 (100%)	*n* = 54 (98%)		
Light (min/week)	0 [0; 0]	0 [0; 0]	0 [0; 0]	0 [0; 0]		
0 min/week	*n* = 76 (100%)	*n* = 71 (100%)	*n* = 58 (100%)	*n* = 55 (100%)		
Moderate (min/week)	0 [0; 0]	0 [0; 0]	0 [0; 0]	0 [0; 0]		
0 min/week	*n* = 76 (100%)	*n* = 71 (100%)	*n* = 58 (100%)	*n* = 54 (98%)		
Vigorous (min/week)	0 [0; 0]	0 [0; 0]	0 [0; 0]	0 [0; 0]		
0 min/week	*n* = 76 (100%)	*n* = 71 (100%)	*n* = 58 (100%)	*n* = 55 (100%)		
Activity score (MET-min/week)	0 [0; 0]	0 [0; 0]	0 [0; 0]	0 [0; 0]		
**Work/school**						
Total (min/week)	0 [0; 0]	0 [0; 0]	0 [0; 0]	0 [0; 0]	0.408	0.373
0 min/week	*n* = 72 (95%)	*n* = 71 (100%)	*n* = 55 (95%)	*n* = 51 (93%)		
Light/moderate (min/week)	0 [0; 0]	0 [0; 0]	0 [0; 0]	0 [0; 0]		
0 min/week	*n* = 73 (96%)	*n* = 71 (100%)	*n* = 55 (95%)	*n* = 51 (93%)		
Vigorous (min/week)	0 [0; 0]	0 [0; 0]	0 [0; 0]	0 [0; 0]		
0 min/week	*n* = 75 (99%)	*n* = 71 (100%)	*n* = 58 (100%)	*n* = 55 (100%)		
Activity score (MET-min/week)	0 [0; 0]	0 [0; 0]	0 [0; 0]	0 [0; 0]		
**Household**						
Total (min/week)	61 [0; 315]	0 [0; 62]	180 [0; 315]	210 [0; 490]	< 0.001^†#‡>¥^	< 0.001^†‡>¥^
0 min/week	*n* = 35 (46%)	*n* = 48 (68%)	*n* = 17 (29%)	*n* = 14 (26%)		
Light/moderate (min/week)	61 [0; 282]	0 [0; 62]	180 [0; 315]	210 [15; 480]		
0 min/week	*n* = 35 (46%)	*n* = 48 (68%)	*n* = 17 (29%)	*n* = 14 (26%)		
Vigorous (min/week)	0 [0; 0]	0 [0; 0]	0 [0; 0]	0 [0; 0]		
0 min/week	*n* = 70 (92%)	*n* = 70 (99%)	*n* = 52 (90%)	*n* = 52 (95%)		
Activity score (MET-min/week)	121 [0; 683]	0 [0; 123]	360 [0; 630]	420 [30; 980]		
**Leisure time** ^c^						
Total (min/week)	65 [0; 214]	300 [105; 613]	368 [121; 700]	420 [160; 1170]	< 0.001^†§#^	0.007^†§#^
0 min/week	*n* = 18 (24%)	*n* = 21 (3%)	*n* = 29 (5%)	*n* = 4 (7%)		
Light (min/week)	0 [0; 0]	0 [0; 0]	0 [0; 0]	0 [0; 0]		
0 min/week	*n* = 74 (97%)	*n* = 70 (99%)	*n* = 55 (95%)	*n* = 53 (96%)		
Moderate (min/week)	0 [0; 105]	210 [35; 630]	245 [70; 693]	420 [70; 1080]		
0 min/week	*n* = 45 (59%)	*n* = 17 (24%)	*n* = 9 (16%)	*n* = 11 (20%)		
Vigorous (min/week)	0 [0; 70]	5 [0; 60]	0 [0; 0]	0 [0; 0]		
0 min/week	*n* = 50 (66%)	*n* = 35 (49%)	*n* = 48 (83%)	*n* = 48 (87%)		
Activity score (MET-min/week)	360 [0; 1185]	1575 [543; 2975]	1580 [534; 2790]	1680 [423; 5040]		
Type of activity (days/week)						
Walking	0 [0; 1] 1.4 (2.6)	0 [0; 0] 1.1 (2.2)	0 [0; 7] 2.6 (3.3)	0 [0; 5] 2.0 (3.0)		
Wheeling	0 [0; 7] 1.9 (3.1)	7 [7; 7] 5.8 (2.6)	7 [0; 7] 4.8 (3.2)	7 [0; 7] 4.3 (3.4)		
Bicycling	0 [0; 0] 0.4 (1.5)	0 [0; 0] 0.8 (1.7)	0 [0; 0] 0.2 (1.0)	0 [0; 0] 0.4 (1.3)		
Handcycling	0 [0; 0] 0.1 (0.4)	0 [0; 1] 0.9 (1.7)	0 [0; 0] 0.3 (1.3)	0 [0; 0] 0.2 (1.0)		
Gardening	0 [0; 0] 0.2 (0.6)	0 [0; 0] 0.0 (0.1)	0 [0; 0] 0.1 (0.3)	0 [0; 0] 0.4 (1.4)		
Odd jobs	0 [0; 0] 0.2 (1.1)	0 [0; 0] 0.2 (1.0)	0 [0; 0] 0.4 (1.6)	0 [0; 0] 0.5 (1.6)		
**Sport** ^c^						
Total (min/week)	0 [0; 28]	90 [20; 150]	43 [0; 120]	0 [0; 60]	< 0.001^†§^	0.003^†^
0 min/week	*n* = 55 (72%)	*n* = 17 (24%)	*n* = 21 (36%)	*n* = 32 (58%)		
Light (min/week)	0 [0; 0]	0 [0; 0]	0 [0; 0]	0 [0; 0]		
0 min/week	*n* = 75 (99%)	*n* = 71 (100%)	*n* = 54 (93%)	*n* = 51 (93%)		
Moderate (min/week)	0 [0; 0]	0 [0; 0]	0 [0; 0]	0 [0; 0]		
0 min/week	*n* = 75 (99%)	*n* = 69 (97%)	*n* = 54 (93%)	*n* = 52 (95%)		
Vigorous (min/week)	0 [0; 20]	90 [0; 150]	30 [0; 94]	0 [0; 30]		
0 min/week	*n* = 56 (74%)	*n* = 19 (27%)	*n* = 25 (43%)	*n* = 37 (67%)		
Activity score (MET-min/week)	0 [0; 195]	720 [185; 1200]	298 [0; 814]	0 [0; 480]		

a Analysed by mixed effect model, incorporating time.

b Analysed by mixed effect model, incorporating time, age, sex, amputation level (low, high), Charlson Comorbidity Index without age, obesity, pain, and undernutrition (well-nourished, moderate or suspected undernutrition/severely undernourished).

c Physical activity during leisure time and sports is described as a separate setting and is not summed.

IQR: interquartile range; min: minutes; MET: metabolic equivalent of task; FTC: failed to converge.

† Significant difference between T1 and T2.

§ Significant difference between T1 and T3.

# Significant difference between T1 and T4.

‡ Significant difference between T2 and T3.

¥ Significant difference between T2 and T4. Significant difference between T3 and T4.

At T1, T2, T3, and T4, 28%, 67%, 71%, and 72% of participants, respectively, met the aerobic component of the WHO PA guidelines of at least 150 min of moderate intensity or 75 min of vigorous intensity per week or an equivalent combination (34).

### Meeting Dutch PA guidelines

At T1, T2, T3, and T4, 16%, 55%, 47%, and 27% of participants indicated that they met the Dutch PA guidelines, which include a minimum of 150 min of moderate to vigorous PA per week and twice a week muscle and bone strengthening activities.

### Activ8

At T1, T2, T3, and T4, 22 (29%), 28 (39%), 29 (50%), and 25 (45%) participants completed the Activ8(s) measurement. Reasons for not completing the Activ8 measurement included: personal reasons such as feeling they had already undergone enough to/on their body, finding it too confronting or useless, feeling spied on, or considering it too much hassle; medical reasons such as surgery on the intact leg or wounds on the intact leg; having multiple changing of wheelchairs; the participant or the post lost the Activ8; not having 2 full days of data, or no reason specified. There were no significant differences in participants’ characteristics or questionnaire-assessed PA between participants completing and not completing the Activ8 measurement per measurement occasion (Table SII).

Accelerometer-derived PA increased across measurement occasions, with median daily PA rising from 5 min at T1 to 54 min per day at T4. The number of active bouts per day ranged between 0.0 [0.0; 0.0] and 2.0 [1.0; 3.0] and fragmentation of active bouts 0.0 [0.0; 0.0] and 0.4 [0.4; 0.5] over measurement occasions. Sedentary time ranged from 1354 to 1400 min per day across all measurement occasions (Table III).

**Table III:** Physical activity per measurement occasion post-amputation as assessed with Activ8(s)

Item	T1 (*n* = 22) Median [IQR]	T2 (*n* = 28) Median [IQR]	T3 (*n* = 29) Median [IQR]	T4 (*n* = 25) Median [IQR]
Number of days Activ8 worn	4.0 (1.5)	4.3 (1.4)	5.0 (1.4)	5.5 (1.0)
Duration of physical activity (min/day)	5 [5; 9]	26 [13; 50]	50 [33; 64]	54 [44; 67]
Walking	3 [1; 7]	2 [0; 5]	0 [0; 24]	0 [0; 27]
Running	0 [0; 0]	0 [0; 0]	0 [0; 0]	0 [0; 0]
Cycling	2 [0; 3]	2 [0; 4]	0 [0; 1]	0 [0; 1]
Manoeuvring/wheeling	0 [0; 0]	21 [0; 45]	25 [0; 56]	45 [0; 61]
Number of active bouts/day	0.0 [0.0; 0.0]	1.0 [0.2; 5.5]	1.5 [0.8; 3.0]	2.0 [1.0; 3.0]
0 active bouts/day	*n* = 18 (82%)	*n* = 6 (21%)	*n* = 1 (3%)	*n* = 2 (8%)
Total duration active bouts (min/day)				
All participants	0 [0; 0]	4 [1; 18]	4 [2; 10]	5 [2; 8]
Participants with active bouts	2 [1; 5] *n* = 4	7 [2; 20] *n* = 22	4 [2; 11] *n* = 28	7 [3; 9] *n* = 23
Average duration active bout (min/day)				
All participants	0 [0; 0]	2 [1; 3]	2 [1; 3]	2 [1; 2]
Participants with active bouts	1 [1; 2] *n* = 4	2 [2; 3] *n* = 22	2 [1; 3] *n* = 28	2 [1; 2] *n* = 23
Fragmentation of active bouts^a^	0.0 [0.0; 0.0]	0.4 [0.1; 0.4]	0.4 [0.4; 0.5]	0.4 [0.4; 0.5]
Number of prolonged active bouts/day	0.0 [0.0; 0.0]	0.0 [0.0; 0.0]	0.0 [0.0; 0.0]	0.0 [0.0; 0.0]
0 prolonged active bouts/day	*n* = 22 (100%)	*n* = 24 (86%)	*n* = 27 (93%)	*n* = 23 (92%)
Standing (min/day)	25 [11; 46]	15 [0; 41]	0 [0; 86]	0 [0; 60]
Sedentary behaviour (min/day)^b^	1400 [1380; 1417]	1391 [1346; 1411]	1354 [1312; 1389]	1370 [1317; 1389]
Number of sedentary bouts/day	14.3 [11.0; 17.6]	21.8 [13.8; 33.5]	30.7 [23.8; 39.0]	32.3 [22.5; 43.0]
Total duration of sedentary bouts (min/day)	1385 [1357; 1408]	1363 [1281; 1391]	1310 [1265; 1362]	1337 [1255; 1356]
Average duration sedentary bouts (min/day)	106 [83; 171]	68 [39; 122]	43 [32; 60]	42 [31; 63]
Fragmentation of sedentary bouts^c^	0.01 [0.01; 0.01]	0.02 [0.01; 0.03]	0.02 [0.02; 0.03]	0.02 [0.02; 0.03]
Number of prolonged sedentary bouts/day	7.8 [6.9; 9.6]	9.0 [7.7; 11.0]	10.3 [9.4; 12.0]	10.5 [9.0; 12.5]
Total duration of prolonged sedentary bouts (min/day)	1314 [1266; 1367]	1217 [1042; 1332]	1109 [1013; 1203]	1129 [923; 1218]
Average duration prolonged sedentary bout (min/day)	175 [142; 213]	137 [103; 179]	102 [89; 128]	96 [85; 132]
Fragmentation of prolonged sedentary bouts	0.006 [0.005; 0.008]	0.008 [0.006; 0.010]	0.010 [0.008; 0.012]	0.010 [0.008; 0.012]
Intensity	n = 21	*n* = 18	*n* = 13	*n* = 12
Active counts/min	1181 [1114; 1279]	1255 [1159; 1364]	1258 [1070; 1375]	1238 [1101; 1514]
Walking counts/min	1181 [1114; 1279]	1255 [1159; 1364]	1258 [1070; 1375]	1238 [1101; 1514]
Overall intensity (counts/min)	46 [35; 61]	81 [62; 96]	81 [65; 114]	105 [56; 118]

a Number of active bouts/duration of active bouts. A higher fragmentation index indicates that time is more fragmented with shorter periods of uninterrupted physical activity.

b Includes lying and sitting over 24 h per day, including sleeping.

c Number of prolonged sedentary bouts/duration of prolonged sedentary bouts. A higher fragmentation index indicates that time is more fragmented with shorter periods of uninterrupted prolonged sedentary behaviour.

IQR: interquartile range; NA: not applicable.

### Factors associated with PA

Factors associated with questionnaire-assessed PA were: age (β = –16 min/week, *p* = 0.009), undernutrition (β = –270 min/week, *p* = 0.013), and grip strength (β = 14 min/week, *p* = 0.014). The associations of questionnaire-assessed PA with age (β = –13 min/week, *p* = 0.055) and undernutrition (β = –211 min/week, *p* = 0.061) became borderline significant in the multivariate model correcting for other covariates. Time and age were among the variables retained in the final multivariate model selected via the Akaike Information Criterion, indicating their relevance to the model fit (Table IV).

**Table IV:** Factors associated with questionnaire-assessed physical activity in minutes per week in univariate analysis, incorporating time, and multivariate analysis

Factor	Univariate^a^			Multivariate^b^			AIC^c^		
	β	Standard error	*p*-value	β	Standard error	*p*-value	β	Standard error	*p*-value
Intercept	476	91	< 0.001	1317	788	0.097	1614	422	< 0.001
Time T1–T2	225	109	0.040	159	113	0.162	224	110	0.043
Time T1–T3	497	116	< 0.001	365	131	0.006	495	117	< 0.001
Time T1–T4	649	118	< 0.001	488	134	< 0.001	630	119	< 0.001
Age (years)	–16	6	0.009	–13	7	0.055	–16	6	0.007
Sex (ref: male)	–54	147	0.715	104	183	0.571			
CCI^c^	–59	34	0.086	–37	34	0.266			
LLA level (ref: low)	–119	150	0.430						
Uni/bilat LLA (ref: uni)	192	175	0.275						
Education (ref: low)*	–9	135	0.948						
Smoking status (ref: current)	14	146	0.925						
Pain*	–1	17	0.974						
Obesity (ref: no)	1	136	0.997						
Undernutrition (ref: no)	–270	108	0.013	–211	112	0.061			
Grip strength*	14	5	0.014	7	8	0.355			

a Analysed by mixed effect model, incorporating time (*n* = 81).

b Analysed by mixed effect model, incorporating time, age, sex, CCI, undernutrition, and grip strength (*n* = 80).

c Analysed using AIC, incorporating time, age, sex, CCI, LLA level, Uni/bilat LLA, education, smoking status, pain, obesity, undernutrition, and grip strength (*n* = 78). ^c^Charlson Comorbidity Index without scoring for age, to avoid overlap with parameter age.

Smoking status at moment of LLA. Current vs never/previous. Pain includes VAS on average in the last week. Undernutrition is based on PG-SGA (dichotomized: well-nourished [ref] vs moderate/suspected undernutrition and severely undernourished).

ref: reference group; CCI: Charlson Comorbidity Index; LLA: lower limb amputation; uni: unilateral; bilat: bilateral.

* *n* = 80 because of missing data.

## DISCUSSION

The current study aimed to determine the PA level at days, weeks, and months post-dysvascular major LLA. The level of questionnaire-assessed and accelerometer-derived PA increases with time after LLA, but it remains at a low level. The intensity of questionnaire-assessed PA is mostly moderate or vigorous, whereas accelerometer-derived overall intensity of PA is mostly relatively low. Most PA is performed during leisure time, and the mode of PA is mostly walking and wheeling. Questionnaire-assessed PA level is negatively associated with age, undernutrition, and positively with grip strength.

Our finding that although PA increased over time, it remained low, aligns with findings in hospitalized people and people after serious orthopaedic injury, e.g., hip fracture, in which activity levels are also low in the days up to years post-injury, both when accelerometer-derived and questionnaire-assessed (35, 36). Similarly, a study in people post-hip fracture found that activity levels increase, but remain low in the days up to months post-hip fracture, also both accelerometer-derived and questionnaire-assessed (37). Low levels of PA following amputation surgery are not surprising, given the immediate physical and psychological impact of the amputation, the recovery process, and the limitations in mobility. In our study, age, undernutrition, and grip strength were identified as factors associated with PA. Moreover, multiple other factors might play a role in PA post-LLA as well, e.g., limited knowledge regarding physical recommendations, limited motivation and self-efficacy, equipment, and skills (4). Sufficient PA is important for cardiovascular health and mental well-being, and reduces the risk of secondary chronic diseases (1–3). Given these benefits and the observed low levels of PA, it is crucial to encourage individuals post-dysvascular major LLA to be more physically active, while addressing their specific challenges.

Remarkably, our study showed that in people with major dysvascular LLA the majority of PA is of moderate or vigorous intensity, based on questionnaire-assessed PA. In other populations, most time is spent on light-intensity activities (38). The intensity of questionnaire-assessed PA was based on a combination of experienced intensity and predefined MET values for age groups. Participants may perceive activities as strenuous as energetic efficiency is reduced post-LLA, meaning that more energy is required for activities when compared with able-bodied persons (39). Additionally, some participants reported engaging only/mainly in therapy sessions, which were generally perceived to be of moderate or vigorous intensity. A considerable proportion of participants, ranging from a quarter to half, depending on the measurement occasion, reported no engagement in light activities. The decrease in perceived vigorous activities observed after 5 weeks post-dysvascular major LLA may be attributed to various factors, including changes in intensity perception due to improved physical functioning, adaptation to living with 1 or no legs, or the use of a prosthesis. Furthermore, many participants stopped therapy before 6 or 9 months post-dysvascular major LLA, which could also contribute to the observed changes in intensity levels. On the contrary, the accelerometer-derived PA, based on active and walking counts, revealed low-intensity PA levels. Relying solely on counts to assess activity intensity ignores the reduced energetic efficiency and thereby may not provide a complete picture of intensity. Incorporating measures such as indirect calorimetry in controlled conditions or heart rate monitoring in day-to-day life could offer a more comprehensive evaluation of activity intensity (40, 41).

Given the challenges for people with dysvascular major LLA (4), PA interventions that are tailored to the specific needs of individuals with LLA are needed. Future research should focus on adapting interventions tailored specifically to people with dysvascular major LLA, and next to assess their effectiveness in improving health, patient, and clinical outcomes. Moreover, our findings suggest the potential importance of addressing nutritional status and grip strength as part of strategies to support physical activity in these populations.

Future research should focus on upgrading MET values for individuals with mobility impairments, as similar activities are likely more energy-intensive for them compared with able-bodied individuals. Developing a compendium of MET values specific to this population would enhance the accuracy of PA assessments. Furthermore, there are PA guidelines for people with a disability (42), regardless of diagnosis. Future studies should aim to increase knowledge on the dose–response relationships between PA and various health and clinical outcomes in individuals with dysvascular LLA specifically, to better tailor PA guidelines to this specific population.

### Study strengths and limitations

The current study has several strengths. Previous studies have primarily focused on the PA of individuals with amputations due to trauma or cancer, often with limited representation of those with vascular causes (5–10). However, it is important to note that 85–95% of all amputations are due to vascular problems (43–45). A key strength of this study is its focus on the dysvascular major LLA population, gaining insight into their PA levels. Another strength is the assessment of PA on multiple measurement occasions, using both accelerometer-derived and questionnaire-assessed measures. Therefore, we not only collected data on duration, intensity, and frequency, but also on mode, to provide a deeper understanding of PA post-dysvascular major LLA.

However, this study also has limitations. A number of participants declined to use the accelerometer. For pragmatic reasons, we reduced the required number of wearing days to include participants’ data, which may have compromised reliability, and we were not always able to ensure representation of both week- and weekend days. If the Activ8 device was not worn on the leg, standing time was automatically recorded as 0 min per day, possibly causing a slight underestimation. Conversely, standing time might have been overestimated if participants slept with a pillow under their leg, causing knee flexion that may have been mistakenly classified as standing. Moreover, due to the limited number of participants wearing the Activ8, no statistical analysis was performed to test changes over time. Lastly, accelerometery assessment of PA behaviour may fail to capture activities involving body parts that are not covered by the device’s placement. Conversely, questionnaire-assessed PA can be prone to individual-level inaccuracies such as under- or overreporting (15), and the intensity of activity may be further biased because MET‑based scoring does not account for the large inter‑individual variability in energetic efficiency among people with LLA (46).

### Conclusion

In the days, weeks, and months post-dysvascular major LLA, the level of PA increases with longer duration post-LLA, but remains low, as assessed by both a questionnaire and an accelerometer. Questionnaire-assessed PA is predominantly of moderate or vigorous intensity, whereas accelerometer-derived PA is mostly of low intensity. Most PA occurs during leisure time, with walking and wheeling being the primary modes. Moreover, age and undernutrition were negatively associated with PA, while grip strength was positively associated. These findings underscore the need for PA interventions for individuals post-dysvascular major LLA, addressing their challenges and supporting people in achieving and maintaining optimal PA levels to improve overall health, patient, and clinical outcomes.

## Supplementary Material



## Trial details

The study was registered on ClinicalTrials.gov with the registration number NCT05747066.
