# Supplementary material for: PHYSICAL ACTIVITY LEVELS ARE LOW IN THE DAYS, WEEKS, AND MONTHS FOLLOWING DYSVASCULAR MAJOR LOWER LIMB AMPUTATION
Source: J Rehabil Med. 2026 May 5;58:44666. doi: 10.2340/jrm.v58.44666 (PMC13182223; doi:10.2340/jrm.v58.44666)
Supplement: Supplementary file 1 [file JRM-58-44666-s1.pdf]

Supplementary material has been published as submitted. It has not been copyedited, or typeset by Journal of Rehabilitation Medicine

Table SI. Assigned MET values for various activities in sports setting.

**Activity**	**MET value**
Fitness, gym, cardiofitness, cardio	5.5
Physical therapy, physical therapy exercises, physical therapy exercises at home, physical therapy weights and cardio, physical therapy (walking exercises and weights), physical and occupational therapy, rehabilitation, gait training, occupational therapy, amputation therapy	5.5
Weightlifting, strength exercises, strength exercises at home	5.5
Assisting transfer from bed to wheelchair and wheelchair to bed using arms/monkey bar	3.0
Gymnastics to music	4.0
Climbing stairs	8.0
Walking	3.5
Road cycling	10.0
Yoga/Pilates, yoga and pilates	4.0
Billiards, pool	2.5
Para-darts	2.5
Arts and crafts	2.0

Table SII. Participants' characteristics of conducting the Activ8 measurement per measurement occasion.

	Activ8 measurement		
	Yes (T1, n=22; T2, n=28; T3, n=29; T4, n=25)	No (T1, n=54; T2, n=43; T3, n=29; T4, n=30)	p-value[a]
Age (years)			
T1	69.8 (7.6)	69.8 (11.7)	0.983
T2	69.0 (12.9)	69.7 (9.8)	0.808
T3	70.1 (9.0)	67.3 (12.9)	0.353
T4	70.4 (9.2)	67.2 (12.7)	0.292
Sex (male)			
T1	55%	80%	0.053
T2	68%	73%	0.908
T3	66%	72%	0.777
T4	64%	80%	0.307
LLA level (low)			
T1	73%	72%	1.000
T2	86%	63%	0.067
T3	83%	72%	0.529
T4	64%	80%	0.976
Adapted-SQUASH (min/week)			
T1	215 [143; 596]	240 [90; 574]	0.950
T2	625 [289; 929]	420 [217; 810]	0.448
T3	780 [315; 1190]	550 [380; 1125]	0.692
T4	900 [585; 1830]	698 [379; 1279]	0.217

[a] T-test, Mann-Whitney U-test, or chi-square.
